# ISL1 predicts poor outcomes for patients with gastric cancer and drives tumor progression through binding to the ZEB1 promoter together with SETD7

**DOI:** 10.1038/s41419-018-1278-2

**Published:** 2019-01-15

**Authors:** Ting Guo, Xian-Zi Wen, Zi-yu Li, Hai-bo Han, Chen-guang Zhang, Yan-hua Bai, Xiao-Fang Xing, Xiao-jing Cheng, Hong Du, Ying Hu, Xiao-Hong Wang, Yong-Ning Jia, Meng-Lin Nie, Meng Xie, Qing-Da Li, Jia-Fu Ji

**Affiliations:** 1 Key Laboratory of Carcinogenesis and Translational Research (Ministry of Education/Beijing), Division of Gastrointestinal Cancer Translational Research Laboratory, Peking University Cancer Hospital & Institute, Beijing, China; 2 Department of Gastrointestinal Surgery, Peking University Cancer Hospital & Institute, Beijing, China; 3 The Tissue Bank, Peking University Cancer Hospital & Institute, Beijing, China; 4 Department of Biochemistry and Molecular Biology, School of Basic Medical Sciences, Capital Medical University, Beijing, China; 5 Department of Pathology, Peking University Cancer Hospital & Institute, Beijing, China

## Abstract

ISL1, a LIM-homeodomain transcription factor, serves as a biomarker of metastasis in multiple tumors. However, the function and underlying mechanisms of ISL1 in gastric cancer (GC) have not been fully elucidated. Here we found that ISL1 was frequently overexpressed in GC FFPE samples (104/196, 53.06%), and associated with worse clinical outcomes. Furthermore, the overexpression of ISL1 and loss-of-function of ISL1 influenced cell proliferation, invasion and migration in vitro and in vivo, including GC patient-derived xenograft models. We used ChIP-seq and RNA-seq to identify that ISL1 influenced the regulation of H3K4 methylation and bound to ZEB1, a key regulator of the epithelial–mesenchymal transition (EMT). Meanwhile, we validated ISL1 as activating ZEB1 promoter through influencing H3K4me3. We confirmed that a complex between ISL1 and SETD7 (a histone H3K4-specific methyltransferase) can directly bind to the ZEB1 promoter to activate its expression in GC cells by immunoprecipitation, mass spectrometry, and ChIP-re-ChIP. Moreover, ZEB1 expression was significantly positively correlated with ISL1 and was positively associated with a worse outcome in primary GC specimens. Our paper uncovers a molecular mechanism of ISL1 promoting metastasis of GC through binding to the ZEB1 promoter together with co-factor SETD7. ISL1 might be a potential prognostic biomarker of GC.

## Introduction

Gastric cancer (GC) is the most frequently occurring malignancy of the gastrointestinal tract in China and certain Southeast Asian populations and the second most common cause of cancer-related death worldwide^1,2^. Metastatic dissemination is a critical determinant of cancer prognosis. Although patients in the early stage of GC can be cured by surgical resection, the overall outcome of patients with GC is very poor due to a high incidence of patients with metastatic disease at the time of diagnosis^3,4^. Although much progress has been made in identifying and characterizing the genetic and epigenetic changes associated with GC, the underlying mechanism of gastric carcinogenesis and metastasis is still poorly understood^5,6^. Thus, searching for molecules that can serve as prognostic markers and/or therapeutic targets of GC remains a priority.

Insulin gene enhancer binding protein 1 (ISL1), a LIM-homeodomain transcription factor, plays an important role in the embryogenesis of pancreatic islets of Langerhans. Mouse embryos with an ISL1 deficiency fail to undergo heart development and neural tube motor neuron differentiation^7–9^. Recently, the role of ISL1 in cancer progression has been gradually recognized and is mainly based on aberrant expression. For example, ISL1 expression is higher in non-Hodgkin lymphoma than in normal lymph nodes or Hodgkin lymphoma;^10^ ISL1 expression is involved in both pancreatic and extrapancreatic neuroendocrine neoplasms^11^. Meanwhile, ISL1 was found to be a novel regulator of the cyclin D1, cyclin B, and c-myc genes in cancer^12,13^. However, the underlying mechanism of ISL1 in gastric carcinogenesis still requires further exploration.

ISL1 was also suggested to be a positive modulator that promotes EMT^14,15^. EMT is a critical regulator of the cancer stem cell (CSC) phenotype, a subpopulation of neoplastic cells with stem cell-like properties, notably the capacity to self-renew and undergo metastasis^16^. EMT-inducing transcription factors (EMT-TFs) can be typically classified into three different protein families, namely, the Snail, ZEB1, and basic helix-loop-helix families^17^. The contribution of the EMT program to the CSC phenotype is thought to be variable and most likely depends on cell type and/or coexisting genetic/epigenetic abnormalities^16^, highlighting the crucial role of abnormal EMT and epigenetic changes in metastasis and tumor relapse.

Epigenetic modification, especially histone methylation, is critical in the tumorigenesis of GC. Many cancers arise from the inappropriate epigenetic effects of misregulated methylation^17^. Changes in histone methylation can either increase or decrease the transcription levels of genes depending on which amino acids on the histones are methylated. For example, methylation of lysine 4 of histone 3 (H3K4) sometimes results in transcriptional activation because this modification enables the DNA to uncoil so that transcription factor proteins and RNA polymerase can access the DNA. In embryonic stem (ES) cells, the promoters of many genes encoding key developmental regulators are associated with both the permissive H3K4me3 and the restrictive H3K27me3 modifications^18^. Active removal of broad H3K4me3 domains by the lysine demethylases KDM5A and KDM5B is required for normal zygotic genome activation and is essential for early embryo development^19^. Targeting the MLL1-H3K4me3 axis is an effective approach to enhance the efficacy of checkpoint immunotherapy against pancreatic cancer^20^. ISL1 promotes the demethylation of tri-methylation of histone H3K27 at the enhancers of Myocd and Mef2c, which are core cardiac transcription factors. ISL1 physically interacts with JMJD3, a H3K27me3 demethylase, ISL1 and JMJD3 partner to alter the cardiac epigenome, instructing gene expression changes that drive cardiac differentiation^21^. However, the underlying mechanism of ISL1 influencing epigenetic modification of gastric carcinogenesis still requires further exploration.

Here, we demonstrate that ISL1 was frequently overexpressed in primary GCs, and its expression was significantly related to metastasis, depth of invasion, and poor outcomes in GC patients. Targeting ISL1 expression with shRNA inhibited the proliferative and invasive capabilities of GC cells as well as the metastatic colonization abilities of GC cells in mouse xenograft models. We further uncovered a novel epigenetic aspect of ISL1’s function, showing that ISL1 impacts the epigenetic status of ZEB1, which is regarded as a master EMT-TF. Notably, depletion of ISL1 prevents methylation of H3K4me3 at the promoter of ZEB1, leading to reduced expression of EMT-TFs. Mechanistically, ISL1 physically interacts with SETD7, a methyltransferase of histone H3K4me3, and are co-recruited to the promoter regions of ZEB1. Moreover, ZEB1 expression was significantly positively correlated with ISL1 and was positively associated with a worse outcome in primary GC specimens. ISL1 might be a potential prognostic biomarker of GC.

## Materials and methods

### Cell culture

GC cell lines NCI-N87, AGS, and HEK293FT were purchased from ATCC (Manassas, VA, USA). The MKN28 cell line was obtained from the Health Science Research Resources Bank (Tokyo, Japan). The BGC823, MGC803, and SGC7901 cell lines were obtained from the Cell Research Institute (Shanghai, China). Cells were cultured in RPMI 1640 medium or high-glucose DMEM (GIBCO, Carlsbad, NY, USA), both of which were supplemented with 10% (v/v) fetal calf serum (GIBCO, NY, USA) and antibiotics, and incubated at 37 °C in a humidified atmosphere containing 5% CO_2_.

### Lentiviral transduction of GC cells

Stable cell lines were established with a lentiviral vector using previously described protocols^22^. Briefly, lentivirus was produced by the co-transfection of HEK293FT cells with lentivirus expression vectors and lentiviral packaging mix (Invitrogen, Carlsbad, CA, USA) according to the manufacturer’s instructions. Specifically, packaging plasmids were co-transfected into HEK293FT cells using Lipofectamine 2000 (Invitrogen, Carlsbad, USA) with short hairpin RNA constructs for targeting ISL1. ISL1-specific shRNA oligos are listed in Supplementary Table 1. Identification of stable cell lines was performed using RT-PCR and western blotting for quantifying the expression levels of ISL1. All the primers are provided in Supplementary Table 2.

### Patients and gastric tissue specimens

A total of 196 paraffin-embedded GC tissues were collected from GC patients who underwent radical gastrectomy at Peking University Beijing Cancer Hospital between January 2003 and December 2007. These patients were tracked until 2012. Additionally, 62 matched GC and adjacent nontumor mucosal tissues (more than 5 cm laterally from the edge of the cancerous region, stored at −70 ℃) were collected from patients undergoing radical surgical resection at Peking University Beijing Cancer Hospital from January 2004 to December 2010. These frozen-tissue patients were tracked until 2015. Overall survival (OS) was calculated beginning from the date of the initial surgery and ending either at the time of death caused by the tumor or at the date of the last follow-up. None of the patients received chemotherapy or radiation therapy prior to surgery. This study was performed with the approval of the Ethics Committee of Peking University Beijing Cancer Hospital, and all the patients signed informed consent forms.

### Immunohistochemistry, western blot, and immunofluorescence

All immunohistochemistry (IHC), western blotting, and immunofluorescence procedures were performed according to protocols described previously^23^. The expression of ISL1 was assessed independently by two experienced pathologists who were blinded to the patients’ clinical outcomes. There was a high level of consistency between the two pathologists, and in the few discrepant cases (<5%), a consensus was reached after joint review. Antibodies used are listed in Supplementary Table 3.

### Proliferation, clone formation, and soft agar assay

Stably transduced GC cells were seeded at a density of 3 × 10^3^ cells/well in 96-well plates, and cell confluence was measured with an IncuCyte Live-Cell imaging system (Essen BioScience). Confluence was determined by the IncuCyte software, based on area (confluence) metrics. All colonies with a diameter >3.5 mm were counted using IPP 6.0 software. All conditions were assessed in triplicate, and three independent experiments were performed.

### Cell migration and invasion assays

Cell migration was assessed with a wound-healing assay. The above cells were seeded at 3.5 × 10^4^ density in 96-well plates, scratch wounds were made simultaneously in all culture wells at 12 h after seeding by using IncuCyte wound maker (Essen BioScience). Scratch wound results were compiled with six wells with one scratch in each well. For the trans-well chamber-based migration and invasion assays, 5 × 10^4^ cells were loaded into an insert, provided with serum-free medium, and allowed to pass through a polycarbonate filter, which had been either pre-coated with 100 μl of matrigel (Becton Dickinson, San Jose, CA) for the invasion assay or left uncoated for the migration assay. The lower chambers were filled with DMEM and 10% FBS. Cells on the upper surface of the filters were wiped out after 24 h (migration assay) or 48 h (invasion assay). The membranes were fixed with methanol for 10 min and stained with 0.5% crystal violet for 10 min. The cells on the underside of the filter were counted in five randomly selected microscopic views.

### In vivo mouse models of GC cell lines

Animal studies were carried out in strict adherence with institutional guidelines. Stable GC cells with depleted endogenous ISL1 (SGC7901 and MGC803) or ectopic expression of ISL1 (MKN28), and their respective control ones were injected into the right hind legs of 5-week-old NOD/SCID mice. Tumor growth was monitored every 3 days by measuring the width and length of the tumors with calipers. The tumor volume was calculated by the formula *V* = *L* × *W*^2^/2.

MGC803/SGC7901cells with or without ISL1 knockdown (1.5 × 10^6^ cells in a 100-μl volume per mouse) were injected into the tail vein of female NOD/SCID mice (5 weeks old) using a 30-gauge needle. Six weeks later, the mice were sacrificed, and the lungs were removed and fixed with picric acid fixative. The presence of lung metastases was evaluated at autopsy. All the studies on mice were conducted according to the National Institutes of Health guide for the care and use of Laboratory animals and approved by the Animal Care and Use Committee of Peking University Cancer Hospital.

### Establishment of a GC patient-derived tumor xenograft (GC-PDX) mouse model

The GC-PDX model was established as previously described^24^. The tumors in mice injected with ISL1-positive cells were sectioned into ~1 mm^3^ pieces, which were infected with lentivirus containing either ISL1 shRNA2#, ISL1 shRNA1#, or Scramble. The pieces were then subcutaneously implanted into the flanks of immunodeficient mice. In contrast, tumors in mice injected with ISL1-negative cells were sectioned as described above and infected with an ISL1 overexpression or control lentivirus.

### Luciferase reporter assay

For the luciferase assay, we generated 1000 bp of the ZEB1 promoter sequence upstream of the first nucleotide of exon 1 (GenBank accession NM_001128128.2). Site-directed mutagenesis of the putative ISL1 binding sites (TAAT>GCCG) in the ZEB1 promoter of these genes was performed using two-step PCR. All transfections were performed as described previously^25^.

### Nuclear extraction, immunoprecipitation, and mass spectrometry

Nuclear extraction and immunoprecipitation experiments were performed as previously described^26^. HEK 293FT cells were transfected with pcDNA3.0-FLAG-ISL1. After 48 h, the cells were lysed, and the resulting lysate was applied to an ANTI-FLAG® M2 Affinity Gel (Sigma-Aldrich A2220). The Gel was then washed and the protein complex was eluted with FLAG peptides (Sigma-Aldrich) followed by LC-MS/MS analysis. Beads were then washed with PBS four times and boiled in 2× SDS sample buffer. The sample was analyzed by SDS-PAGE, followed by Immunoprecipitation. Mass spectrometry was performed as previously described^27^.

### ChIP, ChIP-seq, and RNA-seq

ChIP and ChIP-re-ChIP were performed as previously described^28^. Briefly, cross-linked and isolated nuclei were sonicated using a Diagenode Bioruptor to an average size of ~250 bp for chromatin immunoprecipitation sequencing (ChIP-seq) or ~500 bp for ChIP-qPCR. After pre-clearing with BSA-blocked proteinA/G Sepharose, chromatin was incubated with antibodies at 4 °C overnight. The chromatin immune complexes were recovered with the same BSA-blocked protein A/G beads. For ChIP-seq library construction, cross-linked and isolated nuclei were sonicated to an average size of ~250 bp, and ~1 ng of DNA was prepared as described previously^29^. RNA-seq was performed in SGC7901 cells with overexpression of ISL1. For the RNA-seq libraries, polyA + RNA was isolated using Dynabeads Oligo (dT) 25 (Invitrogen) and constructed into strand-specific libraries using the dUTP method^30,31^.

The ChIP primers used are listed in Supplementary Table 4.

RNA-seq: (https://www.ncbi.nlm.nih.gov/geo/query/acc.cgi?acc=GSE122437)

ChIP-seq: (https://www.ncbi.nlm.nih.gov/geo/query/acc.cgi?acc=GSE122056).

### Data analysis

For ChIP-seq, sequenced reads were aligned to the hg19 genome assembly using BWA. ChIP-seq read density files were generated using Igvtools and viewed in Integrative Genomics Viewer (IGV)^32^. Reads were merged from two biological replicates, and enriched peaks for each ChIP-seq dataset were identified with MACS. RNA-seq sequenced reads were aligned to the GRCh38 genome assembly using Hisat2 (version: 2.0.4). Reads Per Kilobase of exon per Megabase of library size (RPKM) were calculated using a protocol from Chepelev et al.^33^. In short, exons from all isoforms of a gene were merged to create one meta-transcript. The number of reads falling in the exons of this meta-transcript were counted and normalized by the size of the meta-transcript and by the size of the library. GO analysis was conducted with Database for Annotation, Visualization, and Integrated Discovery (DAVID)^34^.

### Statistical analysis

The data are expressed as the mean ± standard deviation (SD). Comparisons between groups were analyzed using Student’s *t*-test or ANOVA, and the Student–Newman–Kleuss method was used to estimate the level of significance. Differences were considered statistically significant at *P* < 0.05.

## Results

### ISL1 was frequently overexpressed in primary GCs and was significantly associated with poor prognosis

**Fig. 1:**
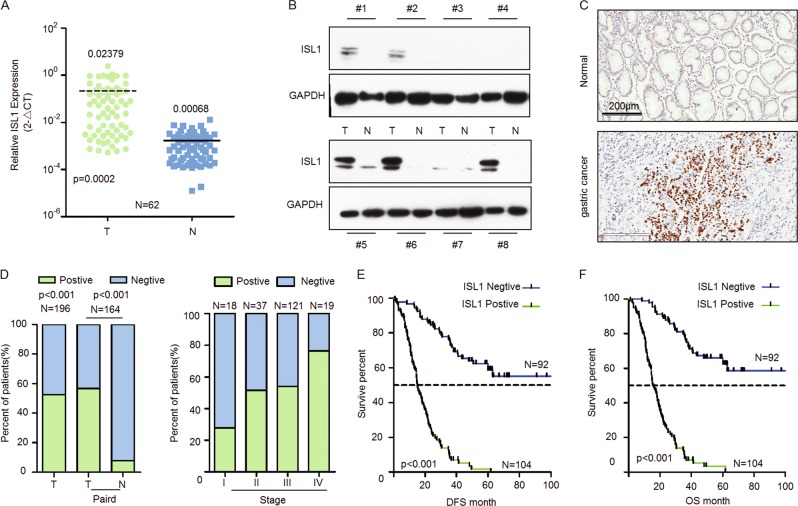
ISL1 expression in primary GC tissues. **a**–**c** The expression of ISL1 in primary GC (*T*) and corresponding surgical margin (*N*) tissues was examined by RT-PCR (**a**), western blotting (**b**), and immunohistochemical staining (**c**). Original magnification: 200× in **c**. **d** Association between ISL1 expression status and TNM stage. **e**, **f** Kaplan–Meier survival curves of disease-free survival (DFS) and overall survival (OS) for patients with ISL1-negative vs. ISL1-positive staining in GC tissues

We assessed the mRNA and protein levels of ISL1 in frozen primary GC tissues by qRT-PCR and western blotting, respectively. As shown in Fig. 1a, ISL1 expression in GC tissues was significantly higher than that in tissues from corresponding surgical margins (0.02379 vs. 0.00068 in median value, approximately 35-fold, *N* = 62). Western blotting also indicated that ISL1 protein expression was upregulated in GC tissues in contrast to matched tissues from the surgical margin (Fig. 1b).

Next, we investigated ISL1 expression in 196 primary GC specimens using IHC. ISL1 was rarely expressed in the normal stomach but was highly expressed in GC tissues and mainly localized to the nucleus of the GC cells (Fig. 1c). As indicated in Fig. 1d and Table 1, the positive staining rate of ISL1 was 53.06% (104/196) in GC samples. In the 164 cases with adjacent noncancerous mucosa from the same section, we observed that ISL1 was more frequently expressed in cancer lesions than in matched noncancerous mucosa samples (56.71% vs. 7.93%, *P* < 0.001; Fig. 1d, left panel).

**Table 1:** Relationship between ISL1 expression and clinicopathological features in patients with gastric cancer

Clinicopathological features	ISL1 expression		*P*-value
	Negative (%)	Positive (%)	
Gender			
Male	65 (46.1%)	76 (53.9%)	0.413
Female	27 (49.1%)	28 (50.9%)	
Age, year			
≤60	48 (51.5%)	46 (48.9%)	0.167
>60	44 (43.1%)	58 (56.9%)	
Primary tumor location			
Non-cardia	77 (49.4%)	79 (50.6%)	0.122
Cardia	15 (37.5%)	25 (62.5%)	
Tumor size (cm)			
≤5	58 (45.6%)	67 (54.4%)	0.479
>5	34 (49.3%)	37 (50.7%)	
Lauren			
Intestinal/mixed	61 (63.9%)	36 (36.1%)	0.001
Diffuse	22 (24.7%)	67 (75.3%)	
Mix	6 (85.7%)	1 (14.35%)	
Vascular invasion			
Absent	61 (74.4%)	21 (25.6%)	0.001
Present	30 (26.8%)	82 (773.24%)	
Histological grade			
Poor	69 (65.7%)	36 (34.3%)	0.001
Well-moderate	22 (24.4%)	68 (75.6%)	
Depth of invasion			
T1 + T2	40 (80.8%)	10 (19.2%)	0.001
T3 + T4	52 (34.7%)	94 (65.3%)	
Lymph-node metastasis			
No	26 (65%)	14 (35%)	0.007
Yes	65 (41.9%)	90 (58.1%)	
Distant metastasis			
M0	90 (50.8%)	87 (49.2%)	0.001
M1	2 (10.5%)	17 (89.5%)	
TNM stage			
I	15 (78.9%)	4 (21.1%)	0.000
II	18 (48.6%)	19 (51.4%)	
III	57 (47.1%)	64 (52.9%)	
IV	2 (10.5%)	17 (89.55%)	

To clarify the role of ISL1 in gastric tumorigenesis, we analyzed the relationship between ISL1 expression and the clinicopathological parameters of GC patients. As depicted in Table 1, GC patients with diffuse type, lymph node metastasis, vascular invasion, and distant metastasis exhibited higher expression levels of ISL1 than those without these characteristics (*P* < 0.001 for all parameters). Likewise, the frequency of ISL1 expression increased remarkably with the progression of TNM stage (*P* = 0.0001, Fig. 1d, right panel). However, there were no significant associations between ISL1 expression and age, gender, tumor location, or tumor size.

Kaplan–Meier survival curves showed that patients with positive ISL1 staining had significantly worse 5-year disease-free survival (DFS, *P* < 0.001; Fig. 1e) and OS (*P* < 0.001; Fig. 1f) than those with negative ISL1 staining. Multivariate Cox regression analysis confirmed that ISL1 expression was an independent prognostic factor for worse OS (*P* = 0.002, Table 2) among GC patients. Similarly, higher mRNA expression of ISL1 indicated worse outcome (*P* = 0.0313, *N* = 62; Fig S1A). The same result was confirmed with the Kaplan–Meier plotter (http://kmplot.com/analysis/; *P* = 0.0051, *N* = 876, Fig S1B). These results suggested that elevated ISL1 expression may serve as a biomarker for poor prognosis of GC patients.

**Table 2:** Results of univariate and multivariate Cox proportional-hazards regression analysis for overall survival of GC patients

Variables	Univariate			Multivariate		
	HR	95% CI	*P*-value	HR	95% CI	*P*-value
Gender	0.861	0.571–1.302	0.479			
Age	1.204	0.842–1.722	0.308			
TNM-stage	2.37	1.686–2.969	0.000	1.958	1.471–2.608	0.000
ISL1 expression	2.880	1.852–4.479	0.000	2.121	1.303–3.451	0.002

### ISL1 altered GC cell growth, migration, and invasion in vitro and in vivo

For the functional study, we examined the expression of ISL1 in different GC cell lines. As shown in Fig. S2, we conducted loss-of-function experiments using SGC7901 and MGC803 cells and gain-of-functional approaches were also performed in MKN28 cells by overexpressing ISL1. The stable cell lines with ISL1 knockdown or overexpression were used for all subsequent in vitro experiments.

**Fig. 2:**
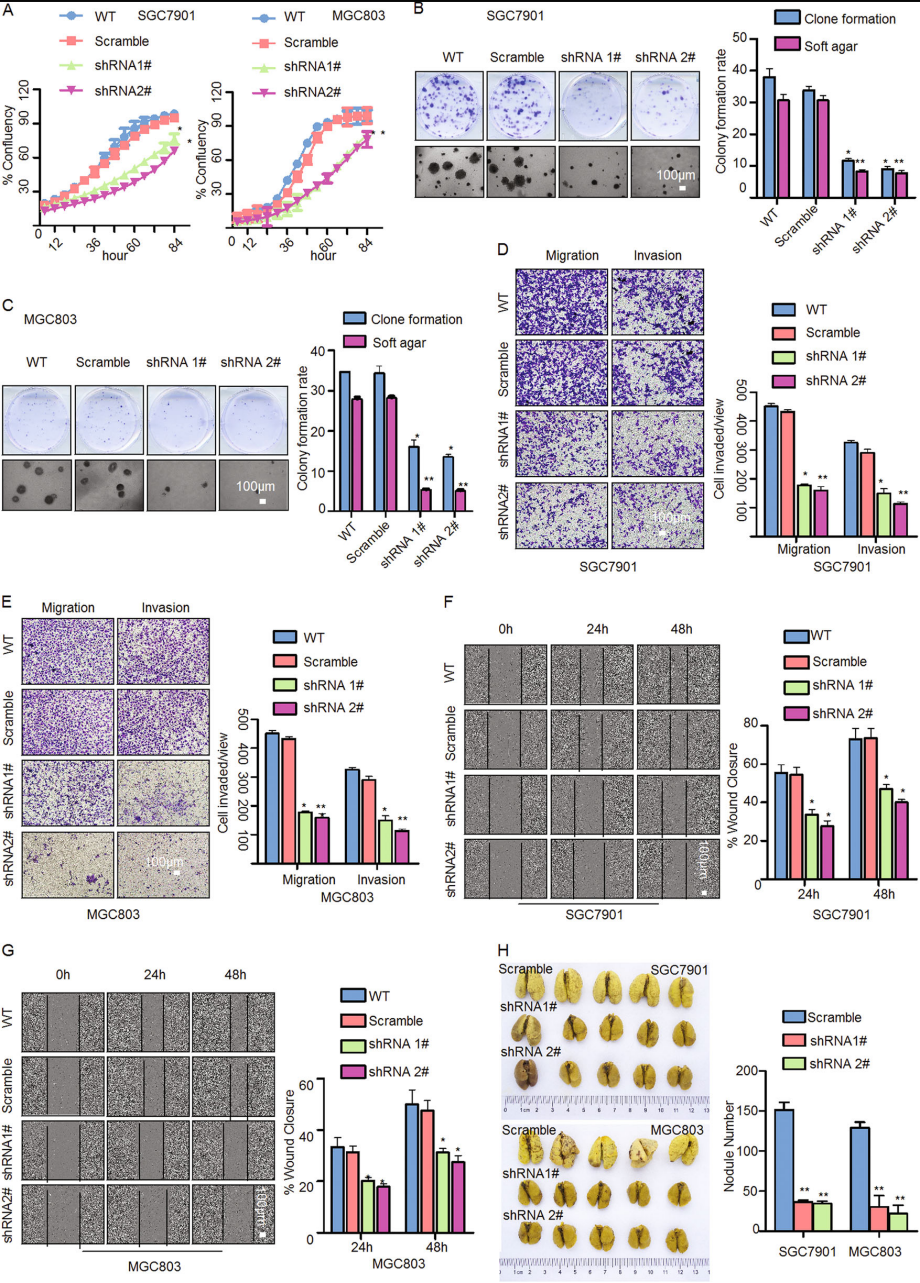
ISL1 knockdown attenuated GC cell growth, migration, and invasion in vitro and in vivo. **a** Cell proliferation was monitored with an IncuCyte system every 6 h. **b**, **c** Plate colony formation assay and soft agar colony formation assay with SGC7901 and MGC803 cells. **d**, **e** Assessment of invasion of SGC7901 and MGC803 cells using Matrigel or Boyden chambers. **f**, **g** Wound healing assay were assayed by live cell imaging on an IncuCyte system. **h** The effect of ISL1 on distant metastatic colonization through blood circulation. Each bar in the bar charts of **a**–**e** represents the mean ± SD from three independent experiments of six replicate wells. *P < 0.05, **P < 0.01. Tumor weights in **h** are shown as the mean ± SD

Using IncuCyte, we determined the effect of ISL1 on GC cell growth in cells with ISL1 knockdown firstly. As shown in Fig. 2a, ISL1 knockdown inhibited cell growth by 38% in SGC7901 cells and by 40% in MGC803 cells at 60 h after knockdown. Meanwhile, ISL1 knockdown visibly reduced colony formation in both SGC7901 and MGC803 cells (Fig. 2b, c). Since ISL1-positive expression is associated with vascular invasion, tumor depth, and lymph node invasion in GC patients, it is possible that ISL1 promotes more aggressive behaviors in GC cells. Indeed, transwell and matrigel invasion assays as well as the wound healing assay showed that both SGC7901-shRNA and MGC803-shRNA (including 1# and 2#) cells displayed decreased migration and invasion compared to their corresponding Scramble cells (Fig. 2d–g, *P* < 0.05). Next, to demonstrate the effect of ISL1 on metastatic colonization, SGC7901-shRNA1#, shRNA2# and MGC803-shRNA1#, shRNA2# cells were introduced into NOD/SCID mice via tail vein injection. The metastatic potential was assessed by counting colonized tumor nodules in the lungs of these mice. The number of tumor nodules in the lungs was reduced by at mostly 80.5% (SGC7901-shRNA2#) and 75.8% (MGC803-shRNA2#) vs. their respective Scramble shRNA (Fig. 2h, *P* < 0.05). Taken together, these data illustrated that ISL1 knockdown strongly reduced cell migration and invasion in GC.

**Fig. 3:**
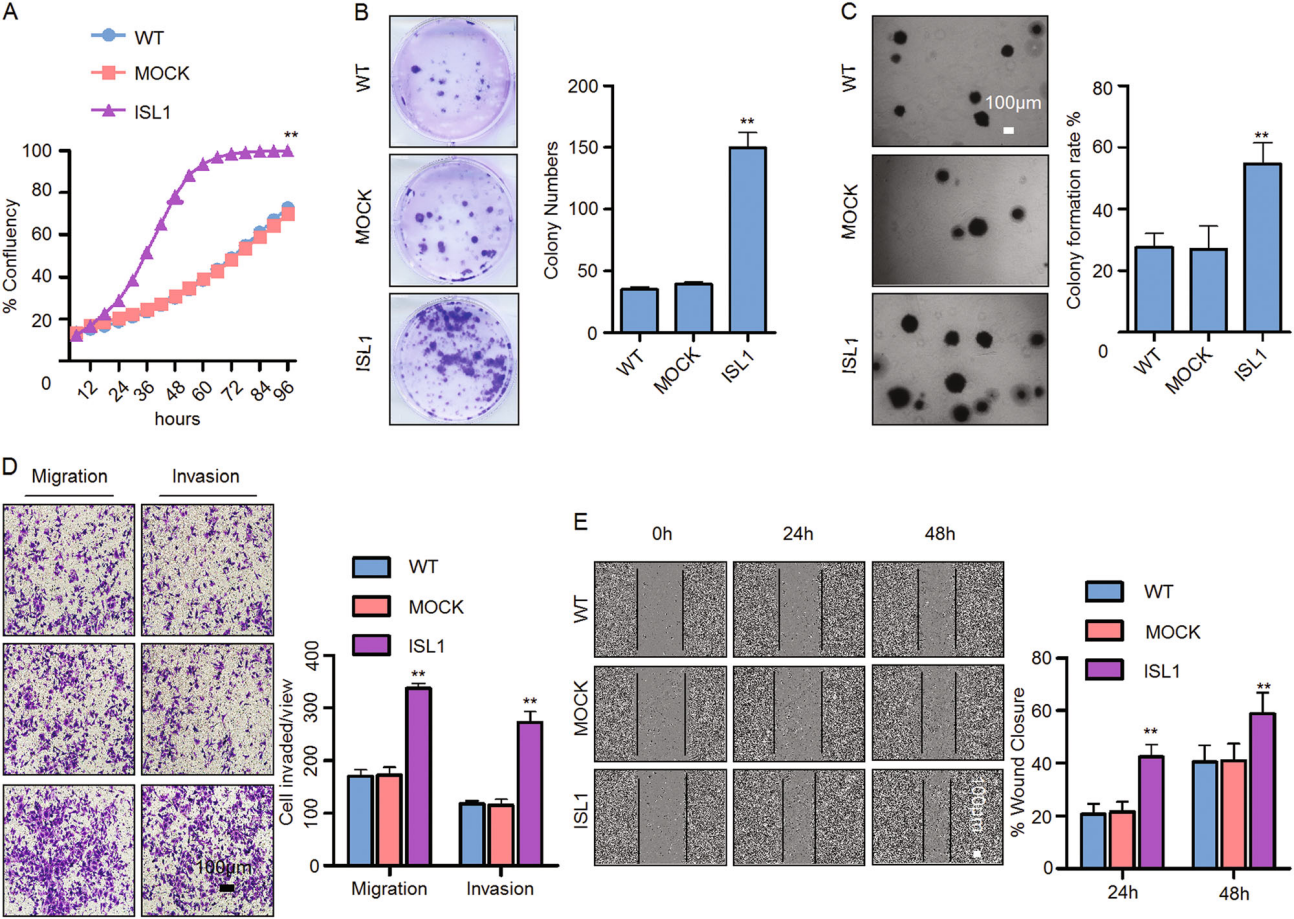
ISL1 overexpression promoted cell growth, migration, and invasion in MKN28 cells. **a** Cell proliferation was monitored with an IncuCyte system every 6 h. **b** Plate colony formation assay. **c** Soft agar colony formation assay. **d** Assessment of invasion of MKN28 cells using Matrigel or Boyden chambers. **e** Wound healing assay by live-cell imaging on an IncuCyte system. Each bar in the bar charts of **a**–**e** represents the mean ± SD from three independent experiments of six replicate wells. *P < 0.05, **P < 0.01

In addition, we performed gain-of-function experiments in MKN28 cells. The results showed that ISL1 overexpression tripled the proliferative abilities of cells after culturing for 60 h as well as enhanced the colony formation, migration, and invasion capacities (Fig. 3).

### ISL1 promoted gastric cell tumorigenesis in vivo

**Fig. 4:**
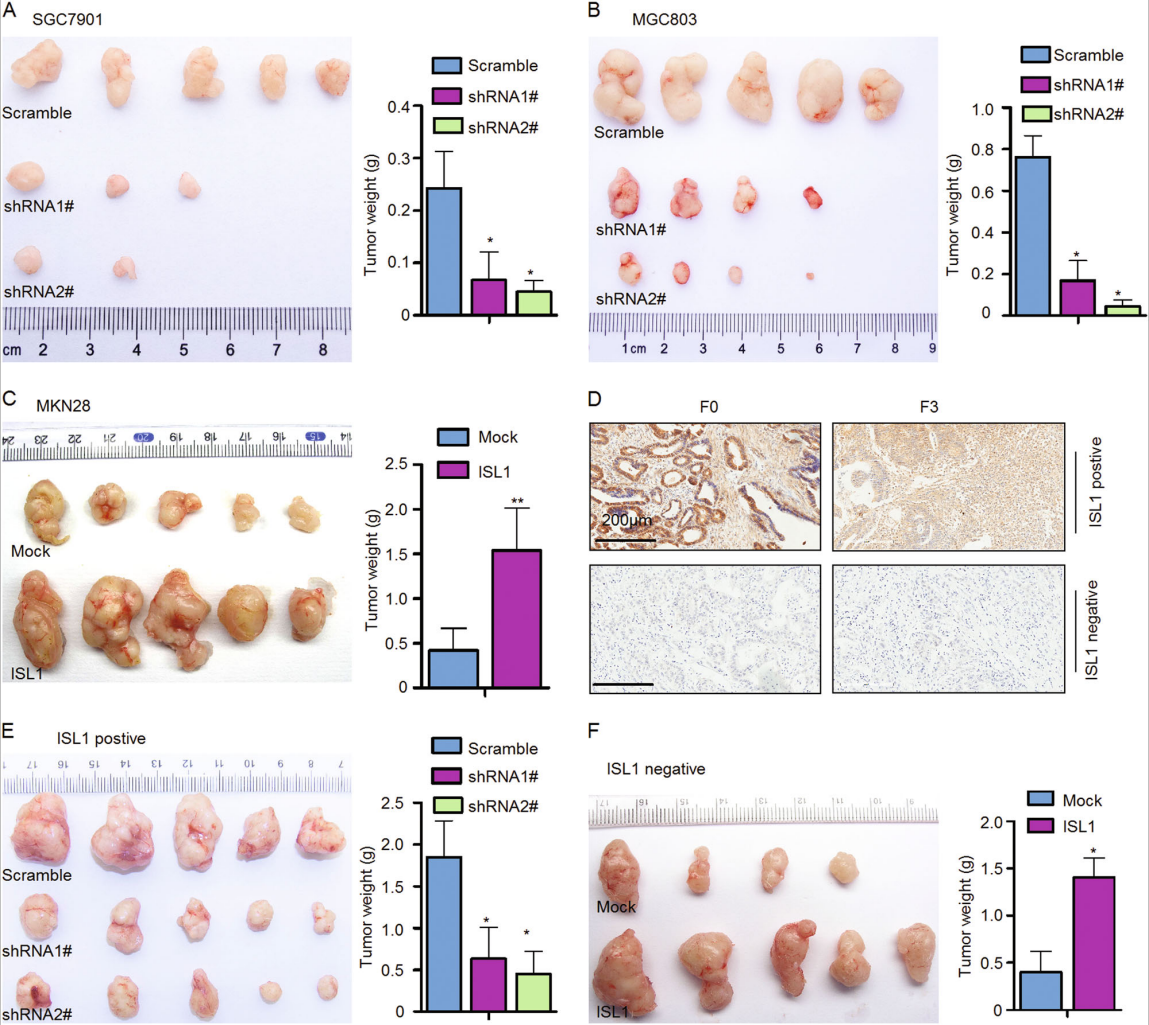
ISL1 promoted tumor growth in cell lines and GC patient-derived xenograft mouse models. **a**–**c** Photographs showing tumor formation in nude mice injected with stable cell lines with ISL1 knockdown (**a** and **b**) and ISL1 overexpression (**c**). **d** Immunohistochemical staining of ISL1. F_0_, primary GC tissue; F_3_, third generation of tumor graft. **e** Photographs showing tumor formation in NOD/SCID mice injected with a mixture of minced tumor graft (F_3_) and lentivirus expressing either ISL1-shRNA1# and ISL11-shRNA2# for ISL1-positive tumor graft (**e**) or ISL1 for ISL1-negative tumor graft (**f**). The tumor volume and weight in **a**–**f** are shown as the mean ± SD. *P < 0.05, **P < 0.01

To understand the effect of ISL1 on GC cell proliferation in vivo, SGC7901-shRNA1#, shRNA2#, MGC803-shRNA1#, shRNA2#, and MKN28-ISL1 (overexpressing) cells were subcutaneously injected into the right hind legs of NOD/SCID mice. In both groups injected with ISL1 knockdown cells (five mice per group), tumor growth was evidently slower, and the resulting tumors were smaller; moreover, two in the SGC7901-shRNA1# group and three mice in the SGC7901-shRNA2# group failed to grow tumors; one mice in the MGC803-shRNA1# and shRNA2# group failed to grow tumors. However, tumor growth was observed in all the mice in the Scramble groups without exception (Fig. 4a, b). Conversely, tumor growth was much more aggressive in the MKN28-ISL1 cell-injected group than that in the corresponding negative control group (Fig. 4c).

Currently, PDX models have been considered a solid preclinical tumor paradigm because they essentially maintain both the genetic and histological features of the primary tumor. Therefore, we used previously established GC-PDX models to evaluate the effect of ISL1 on tumor growth. One ISL1-positive and one ISL1-negative GC-PDX model were assessed by IHC, and the third generation of the xenograft (F_3_) was used in this experiment (Fig. 4d, left panel and right panel). According to our results, mice injected with a mixture of minced tumor graft and shRNA1#, shRNA2# showed an approximately 65%, 75% reduction in average tumor weight compared to mice injected with cells expressing Scramble shRNA (Fig. 4e). Comparatively, mice injected with a mixture of cells with lentivirus-mediated overexpression of ISL1 exhibited a 210% increase in tumor weight compared with mice injected with corresponding control cells (Fig. 4f). Collectively, these data suggest that ISL1 could promote more aggressive malignant features in GC cells in vivo.

### Genome-wide identification of ISL1 targets

**Fig. 5:**
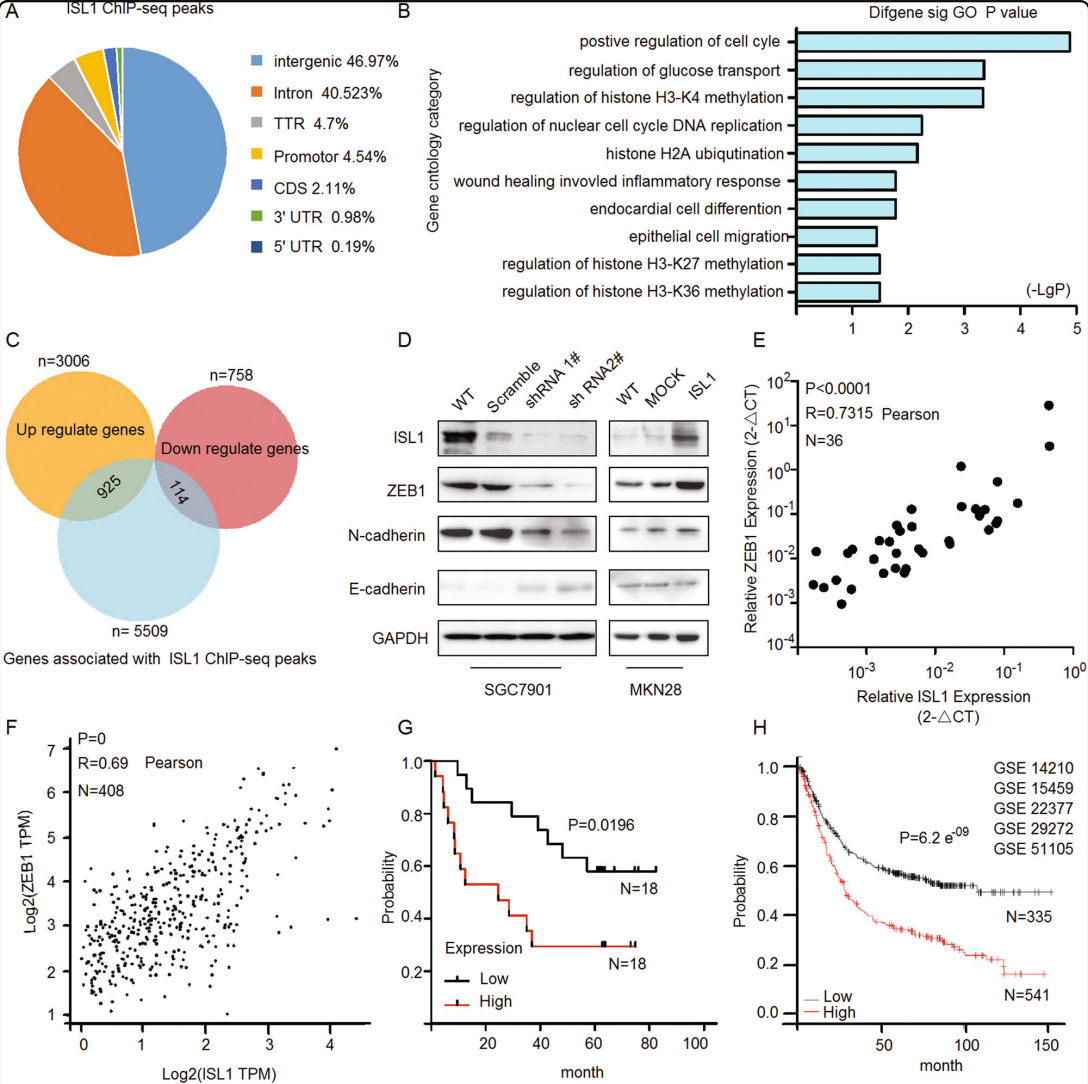
ISL1 enhances gastric cancer tumorigenesis through ZEB1. **a** Pie diagram summarizing the genomic occupancy of ISL1-bound regions as revealed by ChIP-seq. **b** GO functional clustering of upregulated genes allowed the identification of cellular functions directly regulated by ISL1. **c** Genome-wide analysis of downstream targets of ISL1. Overlap of RNA-seq and ChIP-seq results revealed 1039 genes as potential direct targets of ISL1. **d** Western blotting analysis of ZEB1 and EMT-associated proteins. **e** The mRNA expression of ISL1 and ZEB1 was analyzed by real-time RT-PCR in 36 paired GC samples with GAPDH as the reference gene. **f** GEPIA results indicated a correlation between ISL1 and ZEB1 gene expression in stomach adenocarcinoma samples from the TCGA. **g** Kaplan–Meier survival curves of survival time for patients with high vs. normal ZEB1 expression. ISL1 expression was assessed in 36 paired human GC tissues (*P* = 0.0196). **h** Kaplan–Meier survival reanalysis of overall survival. The data were obtained from publicly available gene expression datasets (GSE14210, GSE15459, GSE22377, GSE29272, GSE51105)

To elucidate the mechanisms of ISL1 in gastric carcinogenesis, ChIP-seq and RNA-seq were performed in SGC7901 cells with stable ISL1 overexpression (Fig. 5a–c). Evaluation of differentially bound regions for ChIP-seq data using peaks identified 5509 putative genes (Fig. 5c, blue pie chart), reflecting an average number of 3.2 ISL1-bound regions per gene. The results of RNA-seq revealed a total of 3764 ISL1-responsive genes in which 3006 (Fig. 5c, yellow pie chart) were upregulated (fold change >2, *P* < 0.01) and 758 (Fig. 5c, pink pie chart) were downregulated (fold change <0.5, *P* < 0.01). Comparison and identification of the candidate genes based on the intersection of ChIP-seq and RNA-seq showed that 925 upregulated and 114 downregulated genes had ISL1-bound regions in the Venn diagrams (Fig. 5c). DAVID gene ontology term analysis indicated that these genes were enriched for broad categories of biological processes, especially positive regulation of the cell cycle, regulation of glucose transport (a prerequisite process for tumor invasion), regulation of histone H3K4 methylation, cell migration, and so on (Fig. 5b). Notably, we identified new downstream target genes in 925 upregulated genes, including ZEB1, POU5F1, and MED1 (Fig. S3A), which could be important factors in tumor metastasis and the maintenance of CSCs. We also found that ISL1 could upregulate cyclin D1, cyclin B, and c-Myc (Fig. S3B), which was in agreement with previous reports on pancreatic cancer cells^13^. We also confirmed that ISL1 could upregulate bioinformatics prediction genes (such as DNMT1, SNW1, SETMAR, ASH2L, WHSC1L, SMAD4, MYB) in the regulation of histone H3K4 methylation (Fig. S3C).

### ISL1 enhances gastric cancer tumorigenesis through ZEB1

Tumor cell invasion, dissemination, and metastasis could be triggered by aberrant activation of EMT, and ZEB1 is regarded as a master EMT-TF^17,35^. To gain an insight into the mechanisms underlying ISL1 function in gastric carcinogenesis, we performed a genome-wide analysis and observed that ZEB1 is a possible binding candidate of ISL1. Thus, we analyzed the expression of ZEB1 and other EMT-associated markers in cells with altered ISL1 expression. As shown in Fig. 5d, ISL1 knockdown in SGC7901 cells resulted in downregulation of ZEB1 and N-cadherin and upregulation of E-cadherin, while overexpression of ISL1 in MKN28 cells led to inverse results.

To validate the association between ISL1 and ZEB1, the mRNA expression levels were evaluated by qRT-PCR with GAPDH serving as an internal control in 36 primary GC tissues. Statistical analysis revealed that a significant positive correlation between ISL1 and ZEB1 expression in primary GC exists (Fig. 5e, *R* = 0.73, *P* < 0.0001). The correlation of ISL1 and ZEB1 expression was also analyzed in the public GC dataset from The Cancer Genome Atlas (TCGA, obtained from the GEPIA, Fig. 5f, *R* = 0.69, *N* = 408), and the results further support our above hypothesis. In addition, Kaplan–Meier survival analysis showed that ZEB1 expression was positively associated with a worse outcome in both independent datasets (Fig. 5g, h).

### The ISL1/SETD7 complex directly bound to the ZEB1 promoter with trimethylated histone H3K4

**Fig. 6:**
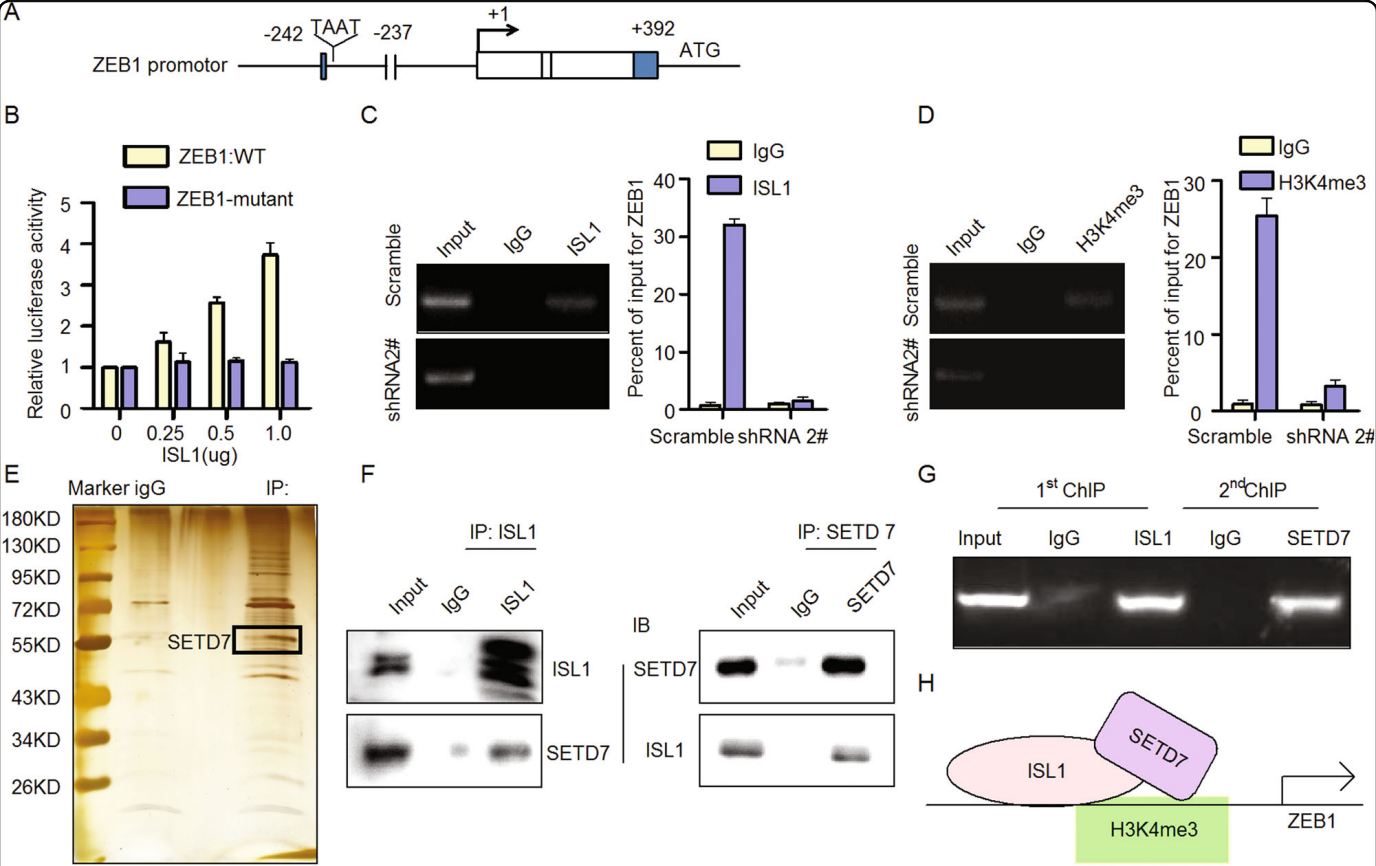
ISL1 promoted ZEB1 expression by H3K4me3 modification through binding SETD7. **a** Consensus binding site (TAAT) for ISL1 on the ZEB1 promotor. **b** Luciferase reporter assay was performed in 293FT cells **c**, **d** Sonicated chromatin from the SGC7901 cells with ISL1 knockdown was immunoprecipitated with ISL1 (**c**) and H3K4me3 antibodies (**d**). The resulting input and ChIP DNA were characterized with qPCR primers specific for ZEB1 genomic loci to calculate the percentage of coprecipitated DNA relative to the input. **e**, **f** Immunoprecipitation and mass spectrometry analysis of cofactors of ISL1. For the coimmunoprecipitation assay, normal IgG served as the negative control. **g** ChIP and re-ChIP assays. The second ChIP was performed using an antibody against SETD7, and the resulting input and ChIP DNA were characterized with qPCR primers specific for ZEB1 genomic loci to calculate the percentage of coprecipitated DNA relative to the input. **h** Schematic depicting how ISL1 activate ZEB1 expression in gastric cancer cells. Each bar in the column chart data of **b**–**d** represents the mean ± SD from three independent experiments in triplicate

To further investigate the interaction between ISL1 and ZEB1, we performed luciferase reporter assays (Fig. 6a). The results showed that ISL1 expression could activate the wild-type ZEB1 reporter in a dose-dependent manner but failed to activate the ZEB1 reporter with a mutation at the predicted ISL1 binding site (Fig. 6b), indicating that ISL1 may bind to the ZEB1 promoter to affect its transcription. ChIP assays indicated that ISL1 could bind to the ZEB1 promoter in SGC7901-Scramble cells (an enrichment of 32-fold), but ZEB1 expression was not detected in SGC7901-shRNA2# cells (Fig. 6c), further suggesting that ISL1 could bind to the ZEB1 promoter to regulate ZEB1 transcription. The ZEB1 reporter assay and ChIP analysis demonstrated that ISL1 could activate ZEB1 by binding to the ZEB1 promoter.

Previous studies have reported that histone H3K4 trimethylation within the ZEB1 promoter was involved in the regulation of ZEB1 in cancer cells^36,37^. Additionally, GO enrichment analysis in Fig. 5b suggested that ISL1 may be involved in the regulation of histone H3K4 methylation. Therefore, we performed ChIP assay using an anti-H3K4me3 antibody followed by ZEB1 promoter-specific qPCR. ZEB1 DNA was detected in SGC7901-Scramble cells but not in SGC7901-shRNA2# cells (Fig. 6d), suggesting that histone H3K4 was trimethylated in the ZEB1 promoter region where ISL1 was bound in SGC7901 cells.

To further investigate how ISL1 may be involved in the regulation of histone H3K4 methylation, we have performed immunoprecipitation and mass spectrometry to identify co-factor of ISL1 (Fig. 6e). Amongst the list of potential candidates of ISL1-interacting proteins, we noted the presence of SETD7, which has been reported as a histone H3K4-specific methyltransferase associated with cancer progression. SET domain proteins maintain gene activity by methylating chromatin to create “codes” of active and repressed gene states^38^. To validate the interaction between ISL1 and SETD7 regulating the state of H3K4me3 on ZEB1 promotor, we performed co-immunoprecipitation analysis (Fig. 6f) and ChIP and re-ChIP assay (Fig. 6g). These results confirmed that SETD7 and ISL1 can bind to form a complex on the ZEB1 promoter in SGC7901 cells. Thus, we suspected that ISL1 interacts with SETD7 to promote ZEB1 expression through H3K4me3 modification by SETD7.

Taken together, these data suggest that the ISL1/SETD7 complex directly binds to the ZEB1 promoter to activate its expression in gastric cells, which consequently leads to cancer tumorigenesis in GC. Considering that SETD7 is a type of lysine methyltransferase and that H3K4me3 is commonly associated with the active transcription of nearby genes^39^, it is possible that the histone H3K4me3 modification in the ZEB1 promoter is mediated by SETD7, which may serve to create a chromatin structure at ZEB1 transcription initiation sites that are more accessible to transcription factors such as ISL1 to promote the initiation of ZEB1 (Fig. 6h).

## Discussion

Aberrant expression of ISL1 plays an important role in tumorigenesis, ISL1 serves as a biomarker of metastasis in pancreatic and extrapancreatic neuroendocrine neoplasms. Here we have shown that ISL1 expression was positively correlated with lymph node metastasis, vascular invasion, distant metastasis, and more advanced TNM stage in GC. Furthermore, elevated ISL1 expression was significantly correlated with poor outcome in GC.

We found that ISL1 transformed the growth and metastasis of GC both in vitro and in vivo, including GC patient-derived xenograft models. Enhanced cell migration and invasion capabilities are important consequences of EMT, an early event in cancer metastasis^40,41^, and play a key role in the tumor progression of various cancers, including GC^36,42–44^. The Snail, ZEB1, and basic helix-loop-helix families compose typical EMT-TFs. Indeed, ChIP-seq and RNA-seq analysis showed that ISL1 influenced the regulation of H3K4 methylation and bound to ZEB1, a key regulator of the EMT. We found that ISL1 influenced the expression of ZEB1 and N-cadherin, genes associated with EMT. Furthermore, ZEB1 expression was significantly positively correlated with ISL1 and positively associated with a worse outcome in primary GC specimens. Given that ZEB1 promotes the stemness and invasiveness of pancreatic and colon cancer cells via activation of EMT^45,46^, our results indicated that the tumorigenic role of ISL1 may be achieved, at least partially, through activation of ZEB1 in GC.

The genome-wide analysis showed that activation of histone modifications in GC cells, especially H3K4-methylation by ISL1, implying that ISL1 could possibly be involved in histone modification. Histone modification plays an important role in gene transcription^39^. Histones modified are important in tumorigenesis and progression in cancer; for example, H3K4me3 modification of the ZEB1 promoter region results in upregulation of ZEB1 and eventually leads to the proliferation and migration of prostate cancer cells^47^. Poised chromatin at the ZEB1 promoter, including H3K4me3 and H3K27me3 modifications, enables cell plasticity and enhances tumorigenicity^36^. We validated ISL1 as activating ZEB1 promoter through influencing H3K4me3 through ChIP. Meanwhile, we confirmed that ISL1 physically interacted with SETD7, a histone H3K4-specific methyltransferase^48^, binding to the ZEB1 promoter to activate its expression in GC cells. However, the mechanism of molecular interactions between ISL1 and SETD7 remain to be determined. Our results indicate that SETD7 represented a cofactor; however, we were unable to rule out whether ISL1 modulated the methylase activity of SETD7. Our genome-wide sequencing analysis showed that ISL1 influenced the regulation of H3K4, H3K27, and H3K36 methylation in GC. We have verified that ISL1 influenced the regulation of H3K4 in GC tumorigenesis. Whether the regulation of histone H3K27 and H3K36 methylation involved in GC tumorigenesis through ISL1 is still unknown.

There were still many other candidate genes identified in our genome-wide sequencing analysis that might also be regulated by ISL1, including cyclin D1, POU5F1, and so on. As shown in Fig S3B, ectopic expression of ISL1 induced cyclin D1, cyclin B, and c-Myc expression in GC cells, which have been previously shown to be involved in cancer cell proliferation in vitro^12,13,49^. POU homeodomain plays a key role in embryonic development and stem cell pluripotency^50,51^. Aberrant expression of POU5F1 in adult tissues is associated with tumorigenesis^52^. Whether POU5F1 is also involved in ISL1-mediated regulation of GC tumorigenesis still requires further investigation. Interestingly, ISL1, as a direct DNMT1 target, hypermethylated and downregulated in mammary tumors and CSCs. DNMT inhibition or ISL1 expression in breast cancer cells limits CSC population^53^. Elevated expression of ISL1 in some human cancers, including pancreatic and prostate cancers, suggesting that the function of ISL1 is tissue and context dependent. Previous reports demonstrated that non-CSCs of human basal breast cancers were plastic cell populations that readily switch from a non-CSC to CSC-state. The observed cell plasticity was dependent on ZEB1, a key regulator of EMT. The ZEB1 promoter converts from a bivalent to active chromatin configuration, ZEB1 transcription increases and non-CSCs subsequently enter the CSC state. We have verified that ISL1 changed the H3K4me3 state at the ZEB1 promoter and ISL1 influenced the expression of ZEB1 and N-cadherin, genes associated with EMT. Whether ZEB1 involved in CSCs relating to ISL1 enhancing GC tumorigenesis needs further study. Interestingly, our genome-wide analyses identified several downstream targets of ISL1 that are involved in metabolic processes. These include MGEA5, PTPN11, and NUP188, potentially involved in the regulation of glucose transport. This is an attractive possibility that requires further experimental validation.

In conclusion, ISL1-positive expression was significantly associated with metastasis and worse outcomes in GC patients. Alteration of ISL1 expression in GC cells influenced cell proliferation, invasion, and migration both in vitro and in vivo. Notably, our study highlights a novel role for ISL1 at the promoter of ZEB1 and provides mechanistic insights into ISL1/SETD7 complex orchestrates histone modification changes, coordinating gene expression driving GC tumorigenesis. Moreover, ZEB1 expression was significantly positively correlated with ISL1 and was positively associated with a worse outcome in primary GC specimens. Our finding suggests that ISL1 could be a critical target gene for the treatment of GC.

## Supplementary information

Figure S1

Figure S2

Figure S3

Supplementary
