# Supplementary figures and images for: ISL1 predicts poor outcomes for patients with gastric cancer and drives tumor progression through binding to the ZEB1 promoter together with SETD7
Source: Cell Death Dis. 2019 Jan 15;10(2):33. doi: 10.1038/s41419-018-1278-2 (PMC6393520; doi:10.1038/s41419-018-1278-2)

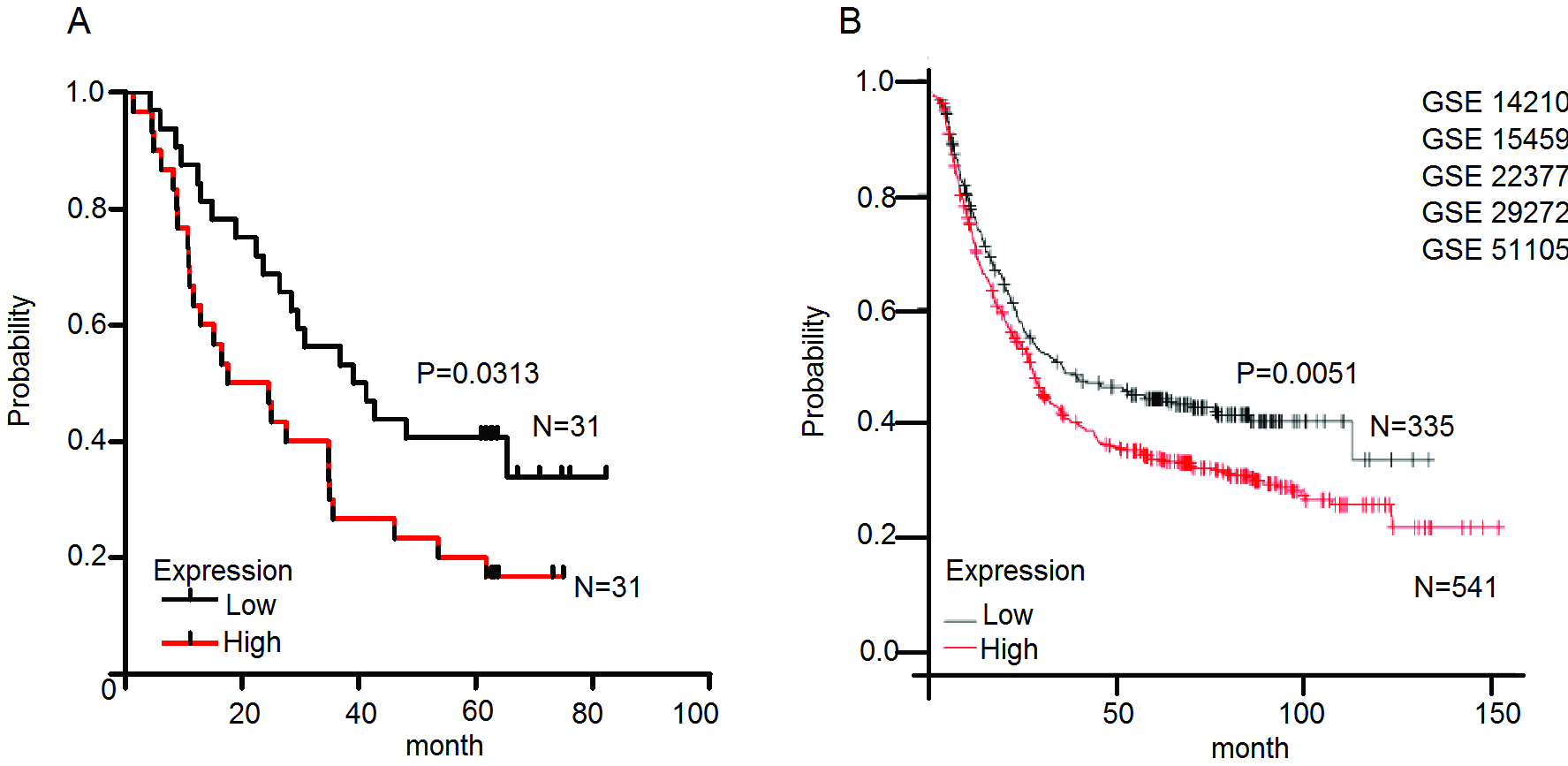

Supplement: Supplementary file 1 — Figure S1 [file 41419_2018_1278_MOESM1_ESM.tif]

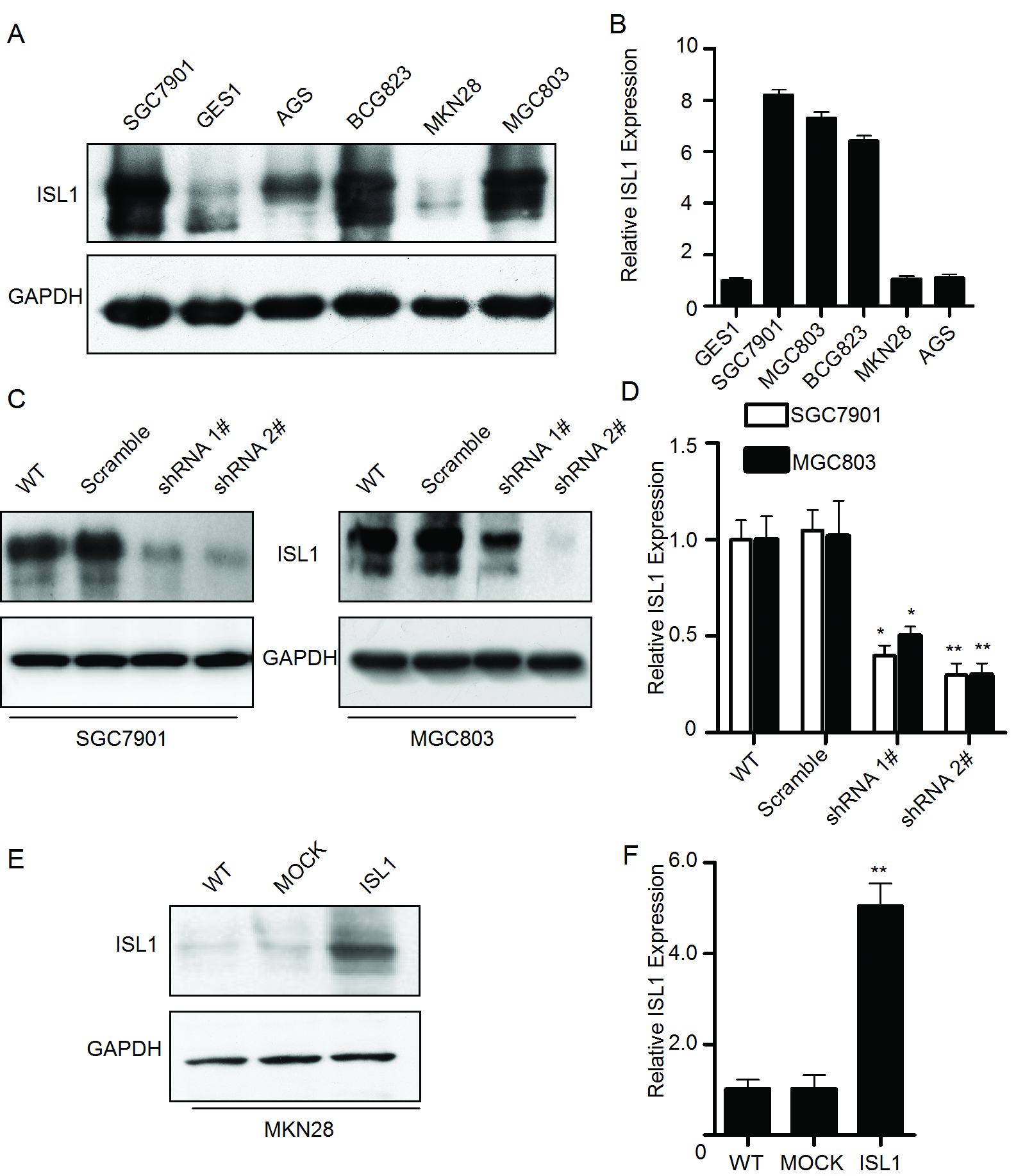

Supplement: Supplementary file 2 — Figure S2 [file 41419_2018_1278_MOESM2_ESM.tif]

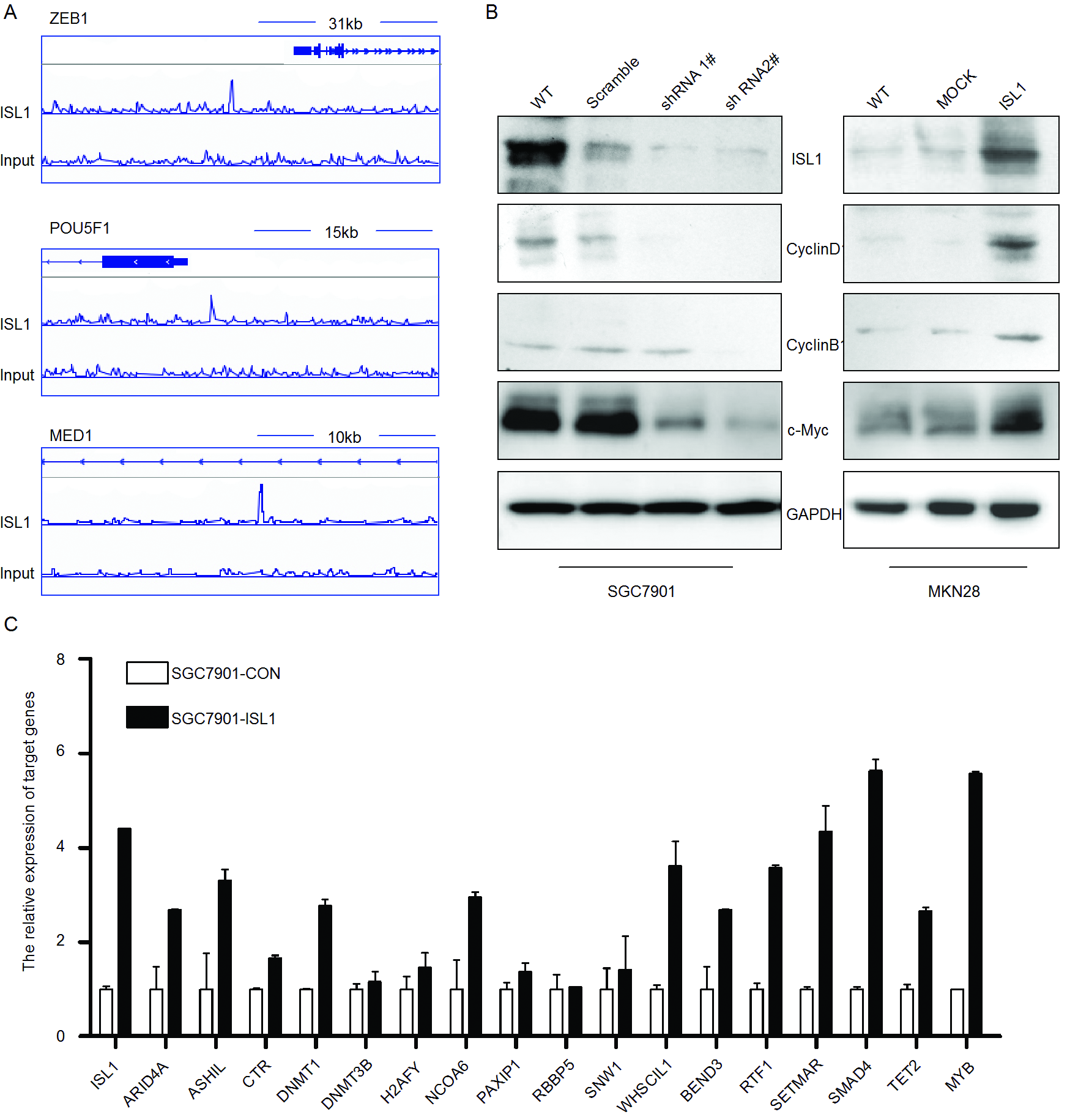

Supplement: Supplementary file 3 — Figure S3 [file 41419_2018_1278_MOESM3_ESM.tif]
