# Supplementary material for: ISL1 predicts poor outcomes for patients with gastric cancer and drives tumor progression through binding to the ZEB1 promoter together with SETD7
Source: Cell Death Dis. 2019 Jan 15;10(2):33. doi: 10.1038/s41419-018-1278-2 (PMC6393520; doi:10.1038/s41419-018-1278-2)
Supplement: Supplementary file 4 — Supplementary [file 41419_2018_1278_MOESM4_ESM.docx]

Supplementary

Table 1: list of shRNA oligos for ISL1:

| **shRNA oligos（5’--- 3’）** | | |
| --- | --- | --- |
| Name | Top Sequence | Bottom Sequence |
| Scramble | GATCCcgtgatcttcaccgacaagatTTCAAGAGAatcttgtcggtgaagatcacgTTTTTC | TCGAGAAAAAcgtgatcttcaccgacaagatTCTCTTGAAatcttgtcggtgaagatcacgG |
| ISL1  shRNA1# | GATCCgagattatatcaggttgtaTTCAAGAGAtacaacctgatataatctcTTTTTC | TCGAGAAAAAgagattatatcaggttgtaTCTCTTGAAtacaacctgatataatctcG |
| ISL1  shRNA2# | GATCCaggagcaactagtggagatTTCAAGAGAatctccactagttgctcctTTTTTC | TCGAGAAAAAaggagcaactagtggagatTCTCTTGAAatctccactagttgctcctG |

Table 2: RT-PCR primers list

| RT-PCR primers | | |
| --- | --- | --- |
| Gene Name | Sense | Anti-sense |
| ISL1 | 5‘-CTGCTTTTCAGCAACTGGTCA-3 | 5’TAGGACTGGCTACCATGCTGT-3 |
| ZEB1 | 5’GCCAACAGACCAGACAGTGTT-3 | 5’TCTTGCCCTTCCTTTCCTG-3 |
| GAPDH | 5'-GACCCCTTCATTGACCTCAAC-3 | 5'-CTTCTCCATGGTGGTGAAGA-3' |
| ASH1L | 5'-ACACTGTCCTTCAAAACGAGAC-3 | 5'-GAAGAGTAGATGGCGTTGCATTA-3 |
| WHSC1L1 | 5'-TTCCAAACACAAGACCACATGA-3 | 5'-ACTCAAACAATTCCCTGCCATTC-3 |
| RBBP5 | 5'-ACTGGAACAAGCAATACCACAG-3 | 5'-GCAGAACCTGCCACGATGTA-3 |
| PAXIP1 | 5'-ATTCTGTCCGTTCAGTGTGGA-3 | 5'-CTTATTGAGGGTTAGCTGGCAA-3 |
| SNW1 | 5'-GGATACCGGAAAGGCTGGATA-3 | 5'-TGACTGTCCTTGTCGAGCAAT-3 |
| NCOA6 | 5'-GTTAGGATGGAGGCGGGATTT-3 | 5'-TTGCTGCTGTATATGGAGCCC-3 |
| DNMT3B | 5'-CCCAGCTCTTACCTTACCATCG-3 | 5'-GGTCCCCTATTCCAAACTCCT-3 |
| H2AFY | 5'-CGGCAGGAACGGTTTTCCA-3 | 5'-TCTCGCTGTCAAAAAGCACGA-3 |
| CTR | 5'-CTCCATCGAGATTCCCCTCC-3 | 5'-TTGCCATCTATACGTGCTGCT-3 |
| DNMT1 | 5'-CCTAGCCCCAGGATTACAAGG-3 | 5'-ACTCATCCGATTTGGCTCTTTC-3 |
| ARID4A | 5'-GACACTTGACCAGCTTCCATT-3 | 5'-GGATACCATTCAGTCCTCTCCG-3 |
| MYB | 5'-ATCTCCCGAATCGAACAGATGT-3 | 5'-TGCTTGGCAATAACAGACCAAC-3 |
| SETMAR | 5'-GAAGCGGCAAAGACGACAC-3 | 5'-GAGTGGGATCAATGTCTGCTC-3 |
| RTF1 | 5'-CGTGTTCCGTTTAGAGTTTGTCT-3 | 5'-TTCATAGCGTAGTTGGGTGGA-3 |
| TET2 | 5'-ATACCCTGTATGAAGGGAAGCC-3 | 5'-CTTACCCCGAAGTTACGTCTTTC-3 |
| BEND3 | 5'-GAACCGTGAGAACAGCTCG-3 | 5'-CTCGTACACGTTTAGCAGGTC-3 |
| SMAD4 | 5'-ACGAACGAGTTGTATCACCTGG-3 | 5'-TGCACGATTACTTGGTGGATG-3 |

Table 3: Antibodies list:

| **Antibodies** | | | |
| --- | --- | --- | --- |
| Name | Company | Catalog # | Applications |
| ISL1 | Abcam | ab109517 | WB, ChIP, |
| ISL1 | Abcam | ab86472 | IHC-P, IP |
| H3K4me3 | Millipore | 17-678 | ChIP |
| IgG | Santa Cruz | sc66931 | IP, ChIP |
| SETD7 | Bethyl | A301-747A | WB, IP, ChIP |
| GAPDH | Abcam | ab8245 | WB, IHC |
| E-cadherin | CST | 3195 | WB, IHC |
| N-cadherin | Sigma | C2542 | WB, IHC |
| ZEB1 | Abcam | ab203829 | WB, IHC |
| Cyclin E1 | CST | 4129 | WB |
| CyclinD1 | CST | 2978 | WB |
| c-Myc | Santa cruze | Sc-40 | WB |

Table 4: ChIP-PCR primers

| Gene Name | Sense | Anti-sense |
| --- | --- | --- |
| ZEB1 | 5‘-TACCTGTTTGTATAATAATGGGCGG -3 | 5’ -TTGCCCCTCTCTTAAAAGTCG 3 |
| H3K4me3 | 5'-CTTGATTCTGAGGGTCAGGAG-3 | 5'-GCTGATGCATAGGTCTGGAAG-3 |

**Supplementary figure legends:**

**Figure S1**: High ISL1 expression was a marker for poor prognosis during GC progression. A: Kaplan-Meier survival curves of survival time for patients with high ISL1 expression *vs*. those with low ISL1 expression in 62 paired human GC tissues (P=0.0313). B: Kaplan-Meier survival reanalysis of overall survival. The data were obtained from publicly available gene expression datasets (GSE14210, GSE15459, GSE22377, GSE29272, GSE51105).

**Figure S2:** ISL1 expression in GC cell lines and the establishment of stable cell lines with ISL1 knockdown or overexpression. A, C, E: Relative ISL1 expression as assessed by western blotting. B, D, F: Relative ISL1 expression as assessed by qRT-PCR. Data in B, D, and F represent the mean ± SD from three independent experiments of triplicate measurement (*P<0.05, **P<0.01 vs. mock or Scramble).

**Figure S3**: Genome-wide downstream targets of ISL1 in GC. A: The binding of ISL1 on the representative target genes ZEB1, POU5F1 and MED1. B: ISL1 expression in cells with stable knockdown (SGC7901) or overexpression (MKN28) of ISL1 was detected by western blotting. C: The relative expression of target genes of the H3K4 methylation in SGC7901-CON and SGC7901-ISL1.
